# Flank pain after minor trauma as the initial manifestation of malignant pheochromocytoma; a case report

**Published:** 2015-06-26

**Authors:** Hassan Niroomand, Atoosa Bagheri-Behzad, Eghlim Nemati, Mehrdad Taghipour, Mohsen Motalebi

**Affiliations:** ^1^Department of Urology, Emam Reza Hospital, Army University of Medical Sciences, Tehran, Iran; ^2^Department of Gynecology, Women’s General Hospital, Tehran University of Medical Sciences, Tehran, Iran; ^3^Nephrology and Urology Research Center, Baqiyatallah University of Medical Sciences, Tehran, Iran

**Keywords:** Flank pain, Pheochromocytoma, Trauma, Hypertension

## Abstract

Pheochromocytoma is a tumor which originates from chromaffin cells of the adrenal medulla or the sympathetic ganglia. This tumor secrets a high amount of catecholamine and metabolites, causing hypertension crisis with headache, tachycardia, sweating and flushing (classic triad of pheochromocytoma). However, in some cases the disease may cause atypical symptoms or may be asymptomatic. The presented patient is a 34-year-old man who referred to our clinic with left flank pain. He had a history of falling from height. In the primary physical examination, a large mass in the abdominal left upper quadrant was palpated. After diagnostic evaluation malignant pheochromocytoma was detected. The patient was discharged on the fourth day after surgery. Malignant pheochromocytoma can presented with atypical symptoms or can be asymptomatic.

**Implication for health policy/practice/research/medical education:**

Pheochromocytoma is a tumor in medulla region of adrenal gland which cause hypertension, headache and tachycardia. The presented patient is a 34-year-old man who referred to our clinic with left flank pain. He had a history of falling from height. In the primary physical examination, a large mass in the abdominal left upper quadrant was palpated. After diagnostic evaluation malignant pheochromocytoma was detected. The patient was discharged on the fourth day after surgery. Malignant pheochromocytoma can presented with atypical symptoms or can be asymptomatic.

## Introduction

Pheochromocytoma is a tumor which originates from chromaffin cells of the adrenal medulla or the sympathetic ganglia. The annual incidence of the disease is 1.6 to 2.1 per million. Around 90% of the cases are sporadic and occur among women. Other cases are young men with a family history and the multiple endocrine neoplasia (MEN) syndrome (1).

Pheochromocytoma can be benign or malignant (2). There is a classic 10 percentage rule for this disease which indicates that 10% of the cases occur in children, 10% is bilateral, 10% is malignant and 10% of it occurs in extra-adrenal sites (1). This tumor secrets high amounts of catecholamines and metabolites, causing hypertension crisis with headache, tachycardia, sweating and flushing (classic triad of pheochromocytoma). However, in some cases the disease may cause atypical symptoms or may be asymptomatic.

## Case presentation

The patient was a 34-year-old man who was referred to our clinic with left flank pain. He had a history of falling from height. In the primary physical examination, a large mass in the abdominal left upper quadrant was palpated and a non-reducing grade III varicocele in left side was detected. His blood pressure was normal, still in some cases during the routine blood pressure examination, it was high. Aside from the occasional abdominal pain, headache, sweating, nausea, weight loss and loss of appetite, the patient’s history and physical examination was not remarkable.

**Figure 1:**
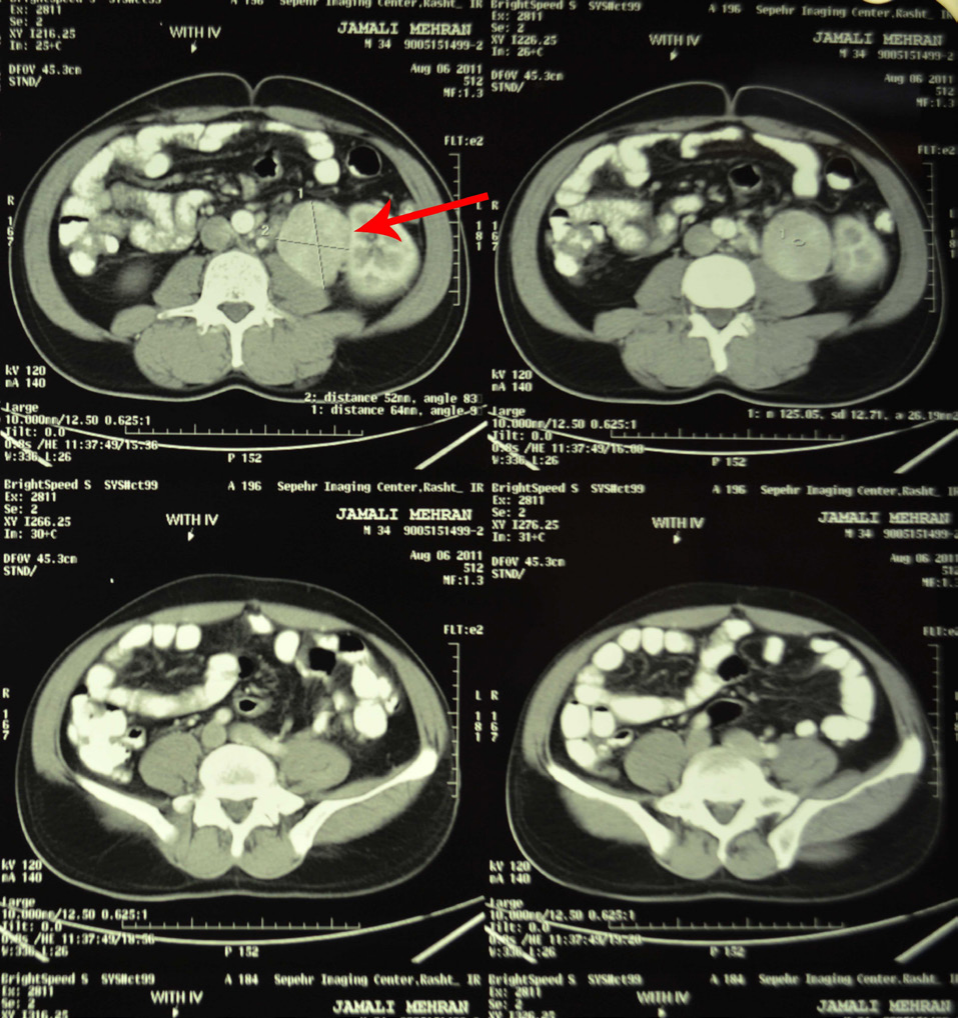
Coronal section of the left adrenal mass which has driven the left kidney downwards.

**Figure 2:**
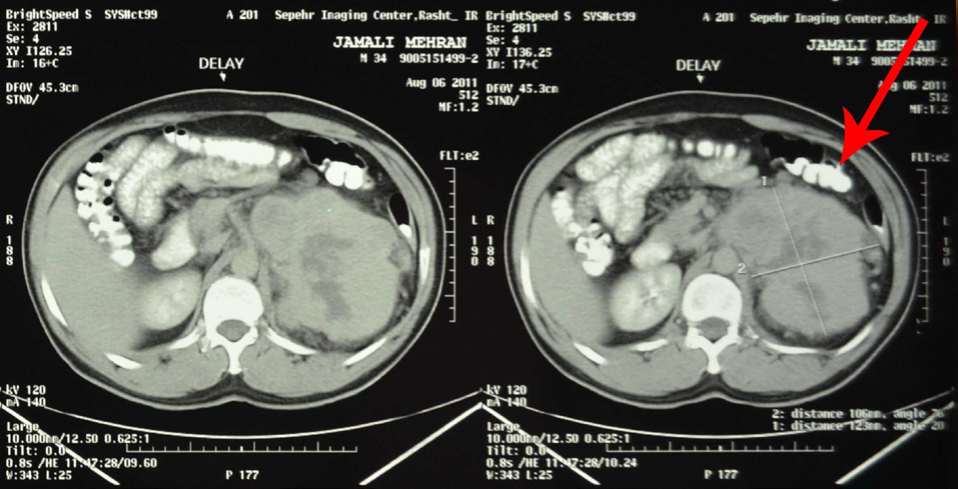
Coronal section of the left adrenal mass which has driven the left kidney downwards.

**Figure 3:**
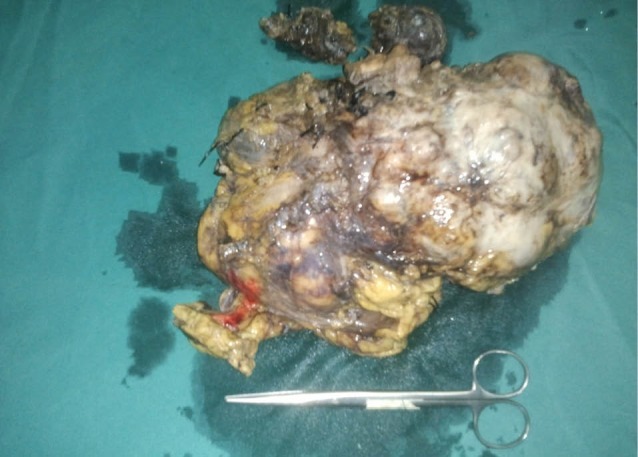
The specimen after surgery.

In ultrasound, a 100×127 mm hypoechoic mass with a central echo free region in the left adrenal and mild hydronephrosis in the left kidney was reported. In computed tomography (CT) scan, there was a 122×107 mm heterodense and lobulated soft tissue mass with central necrosis in the left supra renal region (Figure 1) and another homogenous mass measuring 69×48 mm in the lower level of pararenal space and 30×30 mm mass in the left aorta. In magnetic resonance imaging (MRI), a 120×105×106 mm hyposignal mass in T1w and hypersignal in T2w with heterogenic lobulated margins was seen (Figures 2 and 3). The mass had pressure on the left kidney and pushed it down. Another 131×51×50 mm mass in left para-aortic area medial to the left kidney was also detected.

Liver, spleen, pancreas and other viscera inside the abdomen and pelvis were normal. Metaiodobenzylguanidine (MIBG) scan showed multiple absorptive lesions in the liver and left lung. Echocardiography was performed which was normal. The patient was diagnosed with malignant pheochromocytoma and initially was treated with phenoxybenzamine (60 mg/day) and atenolol for 3 weeks and dextrose saline 3 days prior to surgery. With a subcostal incision, a radical surgery including left adrenal mass removal, left nephrectomy and para-aortic lymphadenectomy was performed (Figure 3). Sodium nitroprusside infusion and three units packed cell were used during the surgery. The patient was monitored in ICU for 48 hours and was discharged on the fourth day.

## Discussion

Pheochromocytoma is a tumor which originates from adrenal chromaffin cells or extra-adrenal (paraganglioma). Its classic symptoms include headache, sweating, tachycardia and paroxysmal hypertension. These symptoms are due to the production and secretion of catecholamine, frequently caused by the tumor. The patient had no objection of any typical cardiovascular symptom. However, following a mild trauma due to falling from low height, he suffered from flank pain and reported a history of headache, sweating, nausea and weight loss in the past months prior to his referral.

In one study it was demonstrated that the classic symptoms of pheochromocytoma are more common among the benign type while backache and abdominal pain are more common in the malignant type (3). The best diagnostic test for patients who are suspected to have pheochromocytoma is always controversial. Detection of clinical symptoms due to overproduction of catecholamine is the major diagnostic step.

Laboratory tests including urine and plasma catecholamine measurement, urinary metanephrine and vanillylmandelic acid (VMA) have a sensitivity of about 76%. Measurement of plasma free metanephrine is the latest available test. Since secretion of catecholamine is periodic and proximal, a single measurement may lead to a false negative. Thus, sensitivity increases with repeated tests (two or three times), particularly following incidence of symptoms (4). Other valuable diagnostic methods include MRI (that reveals 90% of adrenal pheochromocytoma) and MIBG scan. Full abdominal CT scan is valuable in emergency situations (4).

Cytological and histological appearances of benign and malignant pheochromocytoma are often the same. Malignancy is only confirmed by invasion or metastasis (5,6). In our patient the para-aortic lymph node involvement and lung and liver metastasis confirmed the malignancy. Medical treatment preceding elective surgery is the basic treatment. If surgery is performed by a skilled surgeon and an experienced anesthesiologist, the mortality would be less than one percentage (7). A period of 10 to 14 days of medical pre-treatment with alpha receptor inhibitor such as phenoxybenzamine is mostly preferred. Afterwards, the dosage should increase gradually. Adding beta inhibitors (especially in patients who have tachyarrhythmia) prior to surgery is suitable for the preparation.

Other measures include fluids replacement with sugar-salt solutions to prevent orthostatic and postoperative hypotension and hypoglycemia after surgery (8). Any increase in blood pressure during surgery can be controlled by bulbous or continuous infusion of phentolamine, sodium nitroprusside or nicardipine. Tachyarrhythmia can be treated with esmolol infusion (4).

In this disease, surgery and complete tumor resection is the basic and primary treatment. After surgery, the patient should be monitored for 24 hours in ICU. Two major complications after surgery include hypotension and hypoglycemia. Sudden drop in blood pressure caused by the abrupt fall in circulatory catecholamines and hypoglycemia are related to rebound hyperinsulinemia due to recovery of insulin secretion after tumor resection (4).

## Conclusion

Malignant pheochromocytoma can be presented with atypical symptoms or can be asymptomatic. Hence, in case of suspension, physicians should rule out this disease.

## Acknowledgements

We thank Seyed Muhammed-Hussein Mousavinasab for his cooperation in this work.

## Authors’ contribution

All authors contributed equally to the manuscript.

## Conflicts of interest

The authors declared no conflict of interest.

## Ethical considerations

Ethical issues (including plagiarism, data fabrication, double publication) have been completely observed by the authors.

## Funding\Support

None.
